# Longitudinal Follow-Up of Mirror Movements after Stroke: A Case Study

**DOI:** 10.1155/2015/354134

**Published:** 2015-11-15

**Authors:** Hiroyuki Ohtsuka, Daisuke Matsuzawa, Daisuke Ishii, Eiji Shimizu

**Affiliations:** ^1^Department of Physical Therapy, School of Rehabilitation Sciences, Health Sciences University of Hokkaido, 1757 Kanazawa, Ishikari-Tobetsu, Hokkaido 061-0293, Japan; ^2^Department of Cognitive Behavioral Physiology, Chiba University Graduate School of Medicine, 1-8-1 Inohana, Chuou-ku, Chiba 260-8670, Japan; ^3^Research Center for Child Mental Development, Graduate School of Medicine, Chiba University, 1-8-1 Inohana, Chuou-ku, Chiba 260-8670, Japan

## Abstract

Mirror movement (MM), or visible involuntary movements of a relaxed hand during voluntary fine finger movements of an activated opposite hand, can be observed in the hand that is on the unaffected side of patients with stroke. In the present study, we longitudinally examined the relationship between voluntary movement of the affected hand and MM in the unaffected hand in a single case. We report a 73-year-old woman with a right pontine infarct and left moderate hemiparesis. MM was observed as an extension movement of the unaffected right index finger during extension movement of the affected left index finger. The affected right index movement was found to increase, while MM of the unaffected left index finger was observed to decrease with time. These results indicate that the assessment of MM might be useful for studying the process of motor recovery in patients with stroke.

## 1. Introduction

Mirror movement (MM), or visible involuntary movements of a relaxed hand during voluntary fine finger movements of an activated opposite hand, can be observed in the hand that is on the unaffected side of patients with stroke. The hypothesis that acquired MM might be the expression of a compensation process is not new [1] but was never occupied as part of a longitudinal study. Therefore, in this case report, we examined the time-course of changes in the acquired MMs of the unaffected hand. We hypothesized that MMs of the unaffected hand would be altered in the process of the recovery of the affected hand.

## 2. Case Report

**Figure 1:**
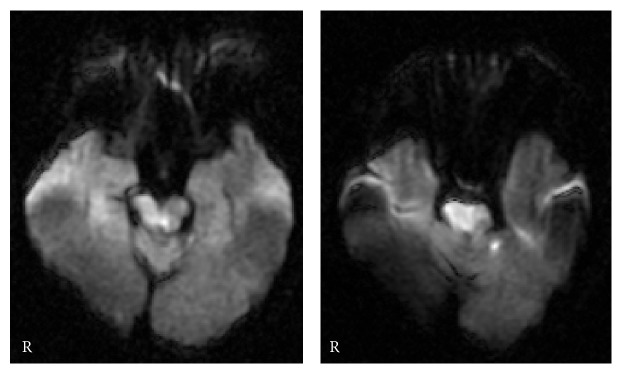
Diffusion-weighted image on the day of stroke onset: lesion in the midbrain and pons.

The subject that participated in this case report was recruited from a long-term health care facility. The subject was a 74-year-old, right-handed woman who had hemiplegia due to a stroke that had occurred nine years before the neurological assessments in this study were conducted. The subject fully understood the purpose and content of this study and gave written informed consent. The subject had no significant past medical history before stroke onset, and a diffusion-weighted image on the day of onset showed a high density area in the ventral side of the midbrain and pons on the right side (Figure 1). At the time of the study, the subject exhibited a normal level of consciousness, and good communication was maintained. The subject's mini-mental state test score was 24/30 and she had mild dysarthria, muscle weakness on the left side of the body, and hypertonus in her left wrist flexor muscles. The subject's deep tendon reflex was increased in her left wrist flexor muscles, and the superficial sensation and deep sensation of her upper limb were normal. Hoffman and Tromner reflexes were present in the subject's left thumb, and MMs could be seen during left upper limb movement (details are described later). The subject displayed no limitation in the range of motion in her left wrist and finger. The Medical Research Council (MRC) scale was used as a parameter of hand motor function: 0, no contraction; 1, palpable contraction, but no visible movement; 2, movement without gravity; 3, movement against gravity; 4, movement against a resistance lower than the resistance overcome by the healthy side; and 5, movement against a resistance equal to the maximum resistance overcome by the healthy side. The MRC scale revealed that the subject's left hand motor function was at a level of 2. Accordingly, the subject underwent physical therapy in 10 minutes per week that involved active assistive range of motion exercise (AAROM) of index extensions of affected finger by a physical therapist. In addition, the subject practiced AAROM as daily voluntary training in 10 minutes per day assisted by the unaffected hand.

In the present study, we quantitatively assessed the subject's voluntary and involuntary movements [2]. In her case, we could see large MMs in the subject's right index finger extension (unaffected side) when she tried to extend her left index finger (affected side). Therefore, we quantitatively monitored the index extension of both sides. To concentrate the subject's attention on her left index extension, her left thumb was held in a steady position by an examiner. Additionally, the subject's right forearm was held in place with a weight so as not to disturb movement. The subject was then instructed to extend her left index finger as far as possible. She was also instructed to watch her left index finger during movement and to keep her right hand at rest. These movements were performed 10 times for three sets at her own pace. A digital camera (Canon IXY digital 70) was used to record the movements of both hands, and the recorded image files were processed on Microsoft Paint software. During left index finger movement, we measured the maximum aperture between the tip of the index finger and the thumb. At that time, we also measured the MM of the right side. We assessed MM at the initial day (Initial), 3 months (3 M), 5 months (5 M), 8 months (8 M), and 13 months (13 M).

**Figure 2:**
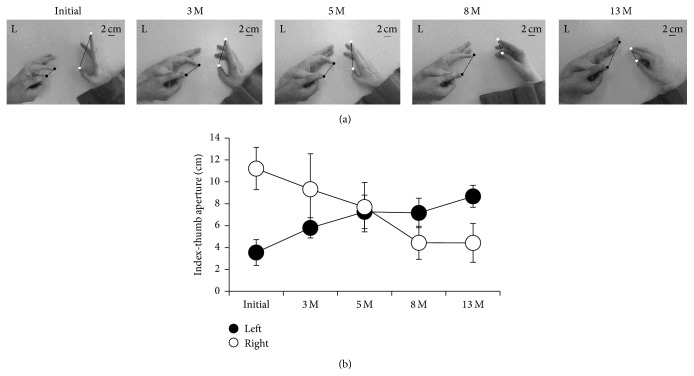
Time-course of changes in MMs during left index finger extension. (a) Typical example of left and right hands during left index finger extension at each time period. The lines connecting the tip of the index finger and thumb indicate the left (filled circle) and right (open circle) aperture. (b) Mean values of the left (filled circle) and right (open circle) aperture at each time period. Error bars indicate SD.

Although the MRC scale maintained the same grade for 13 months, the left index-thumb aperture increased with time.  Figure 2(a) presents a typical example of the left and right hands during left index finger extension at each time period, and Figure 2(b) shows their mean values out of a total of 10 trials. The magnitude of the left index-thumb aperture was smaller than that of the right index-thumb aperture at Initial and 3 M, but larger at 8 M and 13 M. Therefore, while the magnitude of the left index-thumb aperture increased with time, the magnitude of the right index-thumb aperture decreased.

## 3. Discussion

In the present case, we found MMs in a subject's unaffected hand during affected hand movement. Moreover, we found that these MMs decreased with time and that this decrease was concomitant with an increase in affected hand movement. At the beginning of rehabilitation, movement of the subject's affected index finger was limited, and her MM magnitude was large. However, the subject's left index finger movement improved with time, and the magnitude of her MMs became small (Figures 2(a) and 2(b)).

Only two cross-sectional studies have investigated the relationship between motor function of the affected hand and MMs of the unaffected hand [1, 3]. Our findings in the present case are largely in agreement with these previous studies. For example, we demonstrated that there was a close relationship between MMs of the unaffected hand and motor function of the affected hand. Nelles et al. reported that patients with MMs in the unaffected hand exhibited greater motor deficits in the paretic hand than patients without MMs [3]. Furthermore, Kim et al. reported that the magnitude of MMs is correlated with the severity of motor dysfunction [1]. In the present case, we found that MMs of the unaffected hand were much larger when the subject exhibited a more limited range of movement at Initial and 3 M periods; however, MMs of the unaffected finger gradually decreased with increased movement of the affected hand (Figure 2). To our knowledge, the present study is the first to investigate the relationship between motor function of the affected hand and MMs of the unaffected hand in a longitudinal manner.

In previous studies, MMs were observed during effortful finger contraction in healthy subjects [4–6]. The subject in this study had to exert much effort to perform these movements since her baseline MRC score was 2; in other words, the subject was unable to move her index finger against gravity. Further, she had limited motion in her index finger at Initial day and at 3 M. Therefore, MMs in the present case might have been related to the challenging and difficult task. The neural mechanisms underlying motor recovery and acquired MMs involve activation of the ipsilesional and contralesional motor cortices. In the course of motor recovery, it has been reported that activation of the sensorimotor cortex during affected hand movement was shifted from the contralesional side to the ipsilesional side [7]. In the same way, activation of the contralesional sensorimotor cortex is associated with acquired MMs and correlated with MM severity [1]. Recently, Tsuboi et al. provided an animal model of acquired MMs and suggested that enhanced activity in the contralesional motor cortex contributes to MM induction [2]. On the basis of prior studies, MMs in the present case might have been induced by effortful movements as a result of contralesional and ipsilesional cortex activation. Rehabilitation might have then served to improve index finger extension, reducing overactivation of the cortex and decreasing MMs. However, in the present case report, we cannot exclude other neural factors. For example, it is reported that the neural mechanism of congenital MM is the presence of the uncrossed corticofugal fibers, branched bilateral cortico-motoneuronal projections [8]. Further studies using neurophysiological techniques are needed to explain the neural mechanisms of MMs in relation to motor recovery following stroke.

## 4. Conclusion

In conclusion, the present case demonstrated that MMs of the unaffected hand changed with increasing affected hand movement. The present findings indicate that the assessment of MMs might be useful for studying the process of motor recovery following brain damage.

## Supplementary Material

Time-course of changes of voluntary movement of affected hand and mirror movement of unaffected hand. Movie clip shows typical examples of initial day, 5 months and 13 months. Data in Figure 2 were obtained from this movie clip.
